# Taxonomy of the hyper-diverse ant genus *Tetramorium* Mayr in the Malagasy region (Hymenoptera, Formicidae, Myrmicinae) – first record of the *T. setigerum* species group and additions to the Malagasy species groups with an updated illustrated identification key

**DOI:** 10.3897/zookeys.512.9860

**Published:** 2015-07-09

**Authors:** Francisco Hita Garcia, Brian L. Fisher

**Affiliations:** 1 Hessisches Landesmuseum Darmstadt, Friedensplatz 1, 64285 Darmstadt, Germany; 2 California Academy of Sciences, 55 Music Concourse Drive, San Francisco, CA 94118, U.S.A.

**Keywords:** Endemism, Madagascar, taxonomy, *Tetramorium*, *Tetramorium setigerum* species group, zoogeography

## Abstract

In this study we provide an update to the taxonomy of the ant genus *Tetramorium* Mayr in Madagascar. We report the first record of the *Tetramorium setigerum* species group in Madagascar and describe the only Malagasy representative as *Tetramorium cavernicola* **sp. n.**, which is known only from a cave in Ankarana. In addition, we provide an overview of the 19 proposed Malagasy species groups, and discuss their zoogeography and relationships to other groups and larger lineages within the hyper-diverse genus *Tetramorium*. At present, we recognise a highly unique Malagasy *Tetramorium* fauna with 113 species endemic to the island of Madagascar out of a total of 125 translating into an endemism rate of 93%. We hypothesise that this fauna is based on one or a few colonisation events from the Afrotropical region, with subsequent adaptive radiation in Madagascar. Furthermore, we present an updated and illustrated identification key to the *Tetramorium* species groups in the Malagasy region.

## Introduction

The genus *Tetramorium* Mayr, widely distributed throughout all zoogeographical regions, is among the most species-rich ant genera in the world. Currently, we recognise around 600 valid species, but because the authors are aware of a larger number of undescribed species, we expect the total count to be closer to 700 or more species. Most *Tetramorium* species are found in the tropics and subtropics of the Old World, where the genus can be considered hyper-diverse by the definition of Wilson (2003), and are key elements of most local ant communities, especially in the Afrotropical and Malagasy regions. Recent studies in the latter region have revealed an astonishingly diverse and highly endemic *Tetramorium* fauna consisting of 107 valid species plus approximately 17 undescribed species (Hita Garcia and Fisher 2011, 2012a, 2012b, 2014b, unpublished data). Based on these figures, *Tetramorium* is by far the most species-rich ant genus in Madagascar, where it seems to have undergone a radiation that was particularly successful in the forested eastern and northern areas of the island.

On a global scale, Bolton (1976, 1977, 1979, 1980) revised the taxonomy of most regional faunas with the exception of the Palaearctic region. These works provided an excellent foundation for the many later revisions or treatments of species groups/complexes or regions/subregions (e.g. Csösz et al. 2007; Csösz and Schulz 2010; Hita Garcia et al. 2010; Hita Garcia and Fisher 2011, 2012a, 2012b, 2013, 2014a, 2014b; Bharti and Kumar 2012; Sharaf et al. 2012; Vásquez Bolaños et al. 2011). The Malagasy *Tetramorium* fauna was first monographed by Bolton (1979), who treated eight species groups with 36 species (29 of these endemic to Madagascar). The later synonymisation of *Triglyphothrix* Forel (Bolton 1985) under *Tetramorium* added an additional species group with one tramp species; two additional tramp species have been recorded since then (Blard et al. 2003; Roberts and McGlynn 2004). This means that 39 Malagasy *Tetramorium* species were known prior to 2011. In that year, we began a large-scale taxonomic revision of the genus for the Malagasy region based initially on more than 160 morphospecies with more than 40,000 mounted specimens. As a foundation for a series of monographs, we proposed 14 species groups for the Malagasy region and provided a preliminary identification key to these groups (Hita Garcia and Fisher 2011). In addition, we revised the *Tetramorium bicarinatum*, *Tetramorium obesum*, *Tetramorium sericeiventre*, and *Tetramorium tosii* species groups. In that study we described one species and sank another to the rank of junior synonym, which did not change the total species richness for the region. Based on that work, we revised the *Tetramorium bessonii*, *Tetramorium bonibony*, *Tetramorium dysalum*, *Tetramorium kelleri*, *Tetramorium marginatum*, *Tetramorium tortuosum*, *Tetramorium tsingy*, and *Tetramorium weitzeckeri* species groups shortly afterwards (Hita Garcia and Fisher 2012a, 2012b). These studies treated 58 species, of which 45 were described as new, and raised the species count for the region to 84. We also proposed additional species groups leading to a total of 18 for the region. In the most recent study we revised four additional groups: *Tetramorium naganum*, *Tetramorium plesiarum*, *Tetramorium schaufussii*, and *Tetramorium severini* (Hita Garcia and Fisher 2014b). We treated 31 species, of which 22 were newly described, and raised one junior synonym to species status. This increased the current species count for Malagasy *Tetramorium* to 107 (not 106 as mentioned in the introduction of Hita Garcia and Fisher 2014b).

In this study we report the first record of the presence of the Afrotropical *Tetramorium setigerum* species group on Madagascar and describe the single representative in the region as a new species, *Tetramorium cavernicola* sp. n.. With the *Tetramorium setigerum* group, there are now 17 Malagasy species groups that have undergone a current taxonomic revision. Nevertheless, the last two groups, the *Tetramorium ranarum* and the *Tetramorium simillimum* groups, have not been revised since Bolton (1979). The revisions of these two groups are currently in preparation. In addition to the revision of the *Tetramorium setigerum* group, in this study we also present an updated discussion on the currently recognised Malagasy species groups. We give an overview, discuss their biographical affinities, and try to assess their relationships to other key lineages within this hyper-diverse genus. Also, as a consequence of the recent revisions (Hita Garcia and Fisher 2012a, 2012b, 2014b; this study) that proposed additional species groups and added species not known during the preparation of the species group key published in Hita Garcia and Fisher (2011), we present an updated illustrated key to the 19 proposed species groups.

### Abbreviations of depositories

The collection abbreviations follow Evenhuis (2014). The material upon which this study is based is located and/or was examined at the following institutions:

BMNH The Natural History Museum (British Museum, Natural History), London, U.K.

CASC California Academy of Sciences, San Francisco, California, U.S.A.

HLMD Hessisches Landesmuseum Darmstadt, Darmstadt, Germany

MCZ Museum of Comparative Zoology, Harvard University, Cambridge, Massachusetts, U.S.A.

MHNG Muséum d’Histoire Naturelle de la Ville de Genève, Geneva, Switzerland

NHMB Naturhistorisches Museum Basel, Basel, Switzerland

## Material and methods

The material examined for this study and the previous Malagasy *Tetramorium* revisions (Hita Garcia and Fisher 2011, 2012a, 2012b, 2014b) was collected during ant inventories carried out in the Malagasy region from 1992 to 2013. These inventories included material from more than 6,000 leaf litter samples, 4,000 pitfall traps, and 9,000 additional hand collecting events (see Fisher 2005 for additional details). All new type material and all imaged specimens can be uniquely identified by specimen-level codes affixed to each pin (e.g. CASENT0247028). Digital colour montage images were created using a JVC KY-F75 digital camera and Syncroscopy Auto-Montage software (version 5.0), or a Leica DFC 425 camera in combination with the Leica Application Suite software (version 3.8). All images used for the colour plates illustrating the identification key or for the presentation of species are available online and can be seen on AntWeb (http://www.antweb.org). We predominantly have used images of valid species, but in a few cases used images of undescribed species. The latter have morphospecies codes (e.g. *Tetramorium* fhg-forc) and can be seen under their respective codes on AntWeb. The distribution map provided below was generated with R (R Core Team 2014). Morphometric measurements were performed with a Leica MZ 12.5 equipped with an orthogonal pair of micrometres at a magnification of 100×. Measurements and indices are presented as minimum and maximum values with arithmetic means in parentheses. In addition, all measurements are expressed in mm to two decimal places. The measurements and indices used in this study follow Hita Garcia and Fisher (2011, 2012a, 2012b, 2013, 2014a, 2014b):

HL Head length: maximum distance from the midpoint of the anterior clypeal margin to the midpoint of the posterior margin of head, measured in full-face view. Impressions on the anterior clypeal margin and the posterior head margin reduce head length.

HW Head width: width of the head directly behind the eyes measured in full-face view.

SL Scape length: maximum scape length excluding basal condyle and neck.

EL Eye length: maximum diameter of compound eye measured in oblique lateral view.

PH Pronotal height: maximum height of the pronotum measured in lateral view.

PW Pronotal width: maximum width of the pronotum measured in dorsal view.

WL Weber’s length: diagonal length of the mesosoma in lateral view from the posteroventral margin of propodeal lobe to the anteriormost point of pronotal slope, excluding the neck.

PSL Propodeal spine length: in dorsofrontal view the tip of the measured spine, its base, and the centre of the propodeal concavity between the spines must all be in focus. Using a dual-axis micrometre the spine length is measured from the tip of the spine to a virtual point at its base where the spine axis meets orthogonally with a line leading to the median point of the concavity.

PTH Petiolar node height: maximum height of the petiolar node measured in lateral view from the highest (median) point of the node to the ventral outline. The measuring line is placed at an orthogonal angle to the ventral outline of the node.

PTL Petiolar node length: maximum length of the dorsal face of the petiolar node from the anterodorsal to the posterodorsal angle, measured in dorsal view excluding the peduncle.

PTW Petiolar node width: maximum width of the dorsal face of the petiolar node measured in dorsal view.

PPH Postpetiole height: maximum height of the postpetiole measured in lateral view from the highest (median) point of the node to the ventral outline. The measuring line is placed at an orthogonal angle to the ventral outline of the node.

PPL Postpetiole length: maximum length of the postpetiole measured in dorsal view.

PPW Postpetiole width: maximum width of the postpetiole measured in dorsal view.

OI Ocular index: EL / HW × 100

CI Cephalic index: HW / HL × 100

SI Scape index: SL / HW × 100

DMI Dorsal mesosoma index: PW / WL × 100

LMI Lateral mesosoma index: PH / WL × 100

PSLI Propodeal spine index: PSL / HL × 100

PeNI Petiolar node index: PTW / PW × 100

LPeI Lateral petiole index: PTL / PTH × 100

DPeI Dorsal petiole index: PTW / PTL × 100

PpNI Postpetiolar node index: PPW / PW × 100

LPpI Lateral postpetiole index: PPL / PPH × 100

DPpI Dorsal postpetiole index: PPW / PPL × 100

PPI Postpetiole index: PPW / PTW × 100

**Figure 1.**
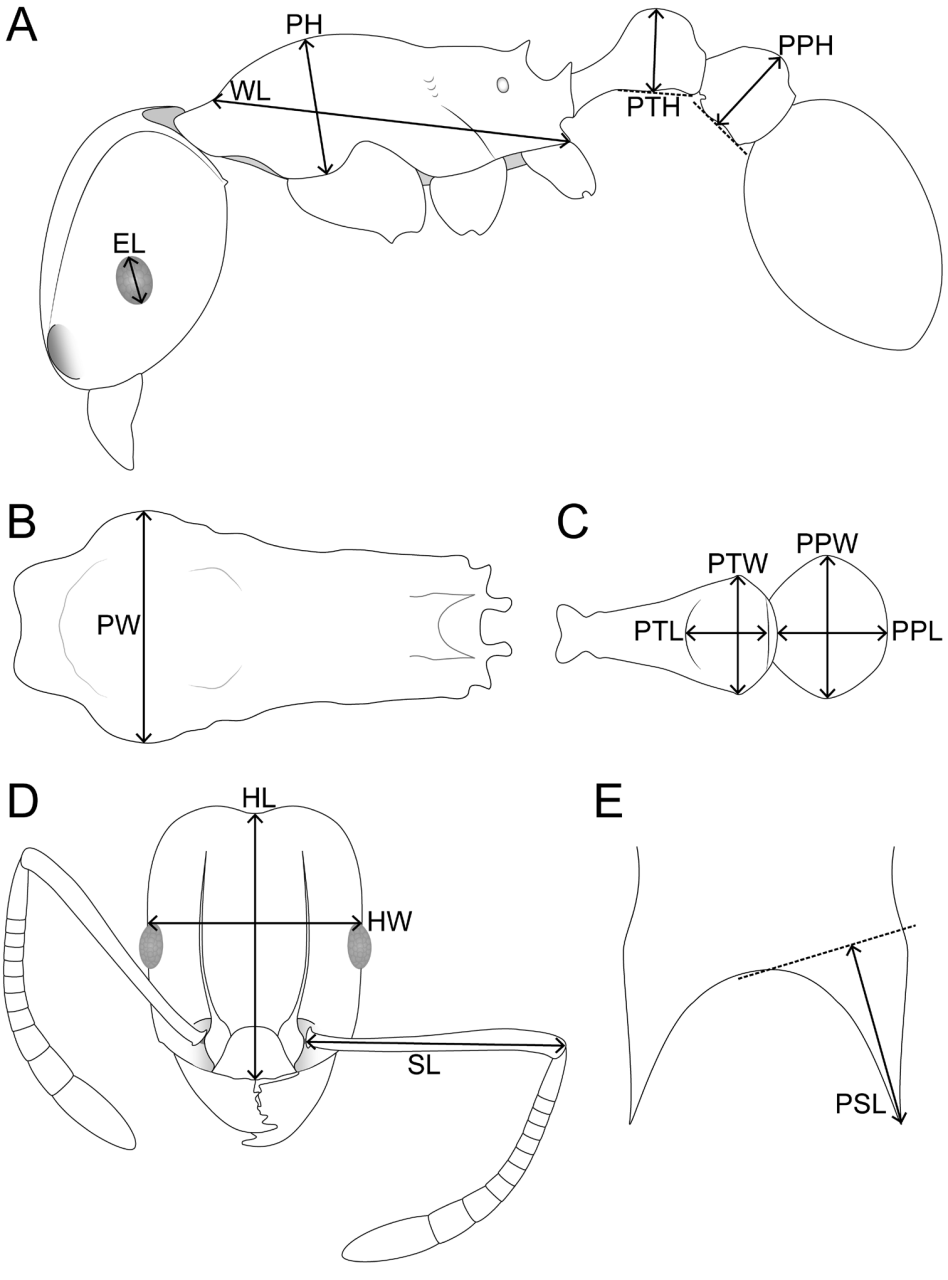
Schematic line drawings of *Tetramorium cavernicola* sp. n. illustrating the used measurements. **A** Body in profile with measuring lines for EL, WL, PH, PTH, and PPH **B** Mesosoma in dorsal view with measuring line for PW **C** Petiole and postpetiole in dorsal view with measuring lines for PTL, PTW, PPL, PPW **D** head in full-face view with measuring lines for HL, HW, and SL **E** Dorsocaudal view of the propodeum with measuring line for PSL.

Pubescence and pilosity are often of high diagnostic value within the genus *Tetramorium* (e.g. Bolton 1976, 1980, 1985; Hita Garcia et al. 2010, Hita Garcia and Fisher 2012a, 2012b). The varying degrees of inclination of pilosity is particularly important for the diagnosis of groups or species. In this context we use the terms “erect”, “suberect”, “subdecumbent”, “decumbent”, and “appressed” following Wilson (1955). The terminology used for the description of surface sculpturing follows Harris (1979) and Bolton (1980).

## Results

### Overview Malagasy species groups

The *Tetramorium* ant fauna of the Malagasy region can be divided into 19 species groups that represent different major lineages within this hyper-diverse genus. However, not all groups are native to the region. A proper assessment of the biogeographical affinities of a region, such as Madagascar and its surrounding South West Indian Ocean island systems, is only possible if comprehensive knowledge on the origin and distribution of each species group is available. This is of special importance when dealing with hyper-diverse genera that possess hundreds of species and dozens of evolutionary lineages throughout most or all zoogeographical regions. Fortunately, in the case of Malagasy *Tetramorium* we are able to assess the whole fauna and classify the groups into native, exotic, or shared with the Afrotropical region. Six of the 19 groups are either completely exotic, contain partly global tramps, or species of African origin. Two of these, the *Tetramorium bicarinatum* and *Tetramorium obesum* groups, are only present in the region with a few very successful global tramp species that very likely originated in the Oriental and Indo-Australian regions (Bolton 1977, 1979; McGlynn 1999; Wetterer 2010; Hita Garcia and Fisher 2011). Four other groups are clearly Afrotropical in origin: the *Tetramorium sericeiventre*, *Tetramorium setigerum*, *Tetramorium simillimum*, and *Tetramorium weitzeckeri* groups. The Malagasy representatives of these groups are either species that have likely been recently transferred from eastern and/or southern Africa by humans, or species that are Malagasy endemics that have evolved from much older colonisation events from Africa, or combinations of both. The *Tetramorium simillimum* group in the Malagasy region is a good example of the latter. It contains two global tramp species of African origin (*Tetramorium caldarium* (Roger) and *Tetramorium simillimum* (Smith), one very widespread non-tramp species of African origin (*Tetramorium delagoense* Forel), as well as some species endemic to the Malagasy region (*Tetramorium anodontion* Bolton, *Tetramorium scytalum* Bolton, and a few undescribed species). The *Tetramorium sericeiventre* group has one species that is widespread in the southern Palaearctic, Afrotropical, and Malagasy regions (*Tetramorium sericeiventre* Emery) and one species endemic to Madagascar (*Tetramorium mahafaly* Hita Garcia & Fisher). The *Tetramorium weitzeckeri* group is represented by *Tetramorium humbloti* Forel, a widespread species distributed in eastern and southern Africa that is probably a more recent introduction to the Malagasy region. The *Tetramorium setigerum* group, here recorded for the first time from the Malagasy region, contains one recently discovered species endemic to Madagascar that we describe below.

Most of the abovementioned, mostly non-native, groups possess twelve-segmented antennae and a triangular to dentiform sting appendage (except the *Tetramorium weitzeckeri* group, which has eleven-segmented antennae and a spatulate sting appendage). The only other Malagasy species group with twelve-segmented antennae is the *Tetramorium tosii* group, which seems to be endemic to Madagascar (Bolton 1979; Hita Garcia and Fisher 2011). In Hita Garcia and Fisher (2011) we discussed the very strong morphological similarities between the *Tetramorium tosii* group and some Afrotropical members of the *Tetramorium setigerum* group, which was not known from Madagascar at the time. At present, we propose to keep both groups separate until more data becomes available, but provide a more thorough discussion in the species group treatment of the *Tetramorium setigerum* group below.

One intriguing finding of the recent revisions (Hita Garcia and Fisher 2011, 2012a, 2012b, 2014b) is that the vast majority of native species groups (*Tetramorium bessonii*, *Tetramorium bonibony*, *Tetramorium dysalum*, *Tetramorium kelleri*, *Tetramorium marginatum*, *Tetramorium naganum*, *Tetramorium plesiarum*, *Tetramorium ranarum*, *Tetramorium schaufussi*, *Tetramorium severini*, *Tetramorium tsingy*, and *Tetramorium tortuosum*) described from Madagascar share the three following key characters: eleven-segmented antennae, anterior clypeal margin notched, and sting appendage spatulate. These twelve groups together contain more than 110 species that are endemic to Madagascar, plus three species found only on Mayotte or the Comoros. The only other group with eleven-segmented antennae, notched anterior clypeal margin, and spatulate sting appendage is the *Tetramorium weitzeckeri* group. However, as noted above, *Tetramorium humbloti* represents a recent arrival from the Afrotropical region and did not evolve independently in Madagascar (Hita Garcia and Fisher 2011). Not considering the latter group, most native species groups could have originated from one or a few ancient colonisation events, with subsequent adaptive radiation that is most pronounced in the humid forests of eastern and northern Madagascar. Assessing the geographic origins of the first colonists with eleven-segmented antennae appears challenging at first glance. A number of species groups have eleven-segmented antennae and a spatulate sting appendage in the Afrotropical, Oriental, and Indo-Australian regions. However, the species morphologically closest to the Malagasy groups are mostly found in Africa.

The most closely related ants seem to belong to the comparatively species-rich *Tetramorium weitzeckeri* species group (Bolton 1980; Hita Garcia et al. 2010). The group is very widespread and ecologically successful in sub-Saharan Africa, and has both eleven-segmented antennae and a spatulate sting appendage. Many of its species resemble a number of members of Malagasy groups, such as the *Tetramorium bessonii* group, parts of the *Tetramorium bonibony* group, parts of the *Tetramorium dysalum* group, and parts of the *Tetramorium marginatum* group. The older species of these groups were initially even placed in the *Tetramorium weitzeckeri* group (Bolton 1979) until recent rearrangements of the Malagasy species group system (Hita Garcia and Fisher 2011, 2012a, 2012b, 2014b). In Hita Garcia and Fisher (2011) we proposed to treat the Malagasy members (with the exception of *Tetramorium humbloti* that stayed in the group) as independent developments from the *Tetramorium weitzeckeri* group and created the new *Tetramorium bessonii*, *Tetramorium bonibony*, *Tetramorium dysalum*, and *Tetramorium marginatum* groups. These groups contain a high degree of morphological diversity often differing from the Afrotropical *Tetramorium weitzeckeri* group. One reason for the distinctiveness of the Malagasy groups was the shape of the petiolar node. We stated that in the *Tetramorium weitzeckeri* group the node is often squamiform with anterior and posterior faces approximately parallel, whereas in Madagascar this form of node is only present in few species of the *Tetramorium dysalum* group. Most other species of the groups in question have petiolar nodes that are anteroposteriorly compressed, often very strongly so, but with a more triangular to cuneiform shape, the node narrowing towards the dorsum, or the anterodorsal margin much more angled than the posterodorsal margin causing the dorsum to strongly taper backward posteriorly. The dorsum of the node is greatly reduced in a number of species, especially from the *Tetramorium bonibony* and *Tetramorium marginatum* groups. Despite being absent in the *Tetramorium weitzeckeri* group, this more triangular node shape is also found in the Afrotropical *Tetramorium squaminode* group, which is also predominantly East and South African. This group also has a spatulate sting appendage but twelve-segmented antennae, and despite this difference seems to be closely related to the *Tetramorium weitzeckeri* group (Bolton 1980; Hita Garcia et al. 2010). Nevertheless, the argument about the difference in node shape is less valid now after the recent description of *Tetramorium mpala* Hita Garcia & Fischer (Hita Garcia and Fischer 2014). This interesting species from Kenya belongs to the *Tetramorium weitzeckeri* group but has a more triangular squamiform node shape like in the Malagasy groups mentioned above. However, instead of reuniting several species groups with more than 70 species, we prefer to wait until more data is available. Morphological similarities in as diverse a genus as *Tetramorium* can be misleading, and better taxonomic resolution on a supraspecific level can only be achieved with a large-scale analysis that combines morphology with informative molecular phylogenetic or phylogenomic data. Regardless, the Malagasy *Tetramorium bessonii*, *Tetramorium bonibony*, *Tetramorium dysalum*, and *Tetramorium marginatum* groups are very likely part of a larger *Tetramorium* lineage that also includes the Afrotropical *Tetramorium weitzeckeri* and *Tetramorium squaminode* groups, even though the relationships remain unclear at present.

The Malagasy *Tetramorium naganum*, *Tetramorium schaufussi*, and *Tetramorium severini* groups (and parts of the *Tetramorium dysalum* group) also appear to have a strong African influence since they share a spatulate sting appendage, high nodiform petiolar node shape, and a lack of any sculpture on the waist segments with the South African *Tetramorium grassii* group, even though the latter group has twelve-segmented antennae. Bolton (1980) stated that the *Tetramorium grassii* group is related and possibly ancestral to the *Tetramorium weitzeckeri* and *Tetramorium squaminode* groups. We concur that these three groups are very likely closely related. Based on their unique morphology, the *Tetramorium plesiarum*, *Tetramorium ranarum*, and *Tetramorium tsingy* groups seem to be independent Malagasy developments since there are no species groups with similar morphology in any region. However, they are very probably also part of the same larger lineage as the other species groups with eleven-segmented antennae and a spatulate sting appendage. This leads us to hypothesise that there is a larger Afrotropical and Malagasy *Tetramorium* clade/lineage that contains the following groups: *Tetramorium bessonii*, *Tetramorium bonibony*, *Tetramorium dysalum*, *Tetramorium grassii*, *Tetramorium marginatum*, *Tetramorium naganum*, *Tetramorium plesiarum*, *Tetramorium ranarum*, *Tetramorium schaufussi*, *Tetramorium severini*, *Tetramorium squaminode*, *Tetramorium tsingy*, and *Tetramorium weitzeckeri* species groups. The situation for the *Tetramorium kelleri* and *Tetramorium tortuosum* groups is less clear. These two groups are very close and were separated recently on the basis of their distinctiveness in Madagascar (Hita Garcia and Fisher 2012b). However, the *Tetramorium tortuosum* group is present in the Neotropical, Afrotropical, Malagasy, Oriental, and Indo-Australian regions, and displays great differences in morphological diversity and species richness from region to region (Bolton 1977, 1979, 1980; Hita Garcia and Fisher 2012b, 2013). It is difficult to assess if all the species now listed as *Tetramorium tortuosum* group indeed belong to one very widespread, possibly very old, monophyletic clade, or some have evolved independently to share several key morphological characters. Consequently, we cannot assess with any certainty whether the Malagasy *Tetramorium kelleri* and *Tetramorium tortuosum* groups are more closely related to the African or Asian members of the *Tetramorium tortuosum* group, or represent a more independent lineage.

In summary, we were able to identify a highly unique Malagasy *Tetramorium* fauna. We recognise 12 of the 19 species groups and an astonishing 113 of the 125 species as Malagasy endemics. This results in an endemism rate of 93%, which is more or less in agreement with the published value for the whole Malagasy ant fauna (ca. 96% in Fisher 2003). In Table 1 we provide an overview of the Malagasy species groups with data on their biogeography, key characters, taxonomic revisions, and preferred habitats.

**Table 1.** Overview of all 19 Malagasy species groups recognised in this study. For each group we provide number of Malagasy species, zoogeographical affinities, number of antennal segments, shape of sting appendage and anterior clypeal margin, the last taxonomic revision, and habitat preferences. The following abbreviations are used for zoogeographical affinities: AFR=Afrotropical, INA=Indo-Australian, MAD=only Madagascar, MAL=Malagasy (Madagascar plus islands of the Southwest Indian Ocean), NEA=Nearctic, ORI=Oriental, T=panglobal tramp.

**Number Malagasy spp.**	**Species group name**	**Zoogeography**	**Antennal segments**	**Sting appendage**	**Anterior clypeal margin**	**Recent taxonomic revision**	**Habitat preferences**
6	***bessonii***	MAD	11	spatulate	notched	Hita Garcia and Fisher 2012a	dry forests, savanna, grassland, anthropogenic habitats
8	***bonibony***	MAD	11	spatulate	notched	Hita Garcia and Fisher 2012a	dry forests, savanna, grassland, anthropogenic habitats
10	***dysalum***	MAD	11	spatulate	notched	Hita Garcia and Fisher 2012a	predominantly lowland or montane rainforests
2	***kelleri***	MAL	11	spatulate	notched	Hita Garcia and Fisher 2012a	dry and humid forests
6	***marginatum***	MAD	11	spatulate	notched	Hita Garcia and Fisher 2012a	lowland or montane rainforests
5	***naganum***	MAD	11	spatulate	notched	Hita Garcia and Fisher 2014	lowland or montane rainforests
5	***plesiarum***	MAD	11	spatulate	notched	Hita Garcia and Fisher 2014	dry forests, savanna, grassland
21	***ranarum***	MAD	11	spatulate	notched	in preparation	predominantly lowland or montane rainforests
20	***schaufussi***	MAL	11	spatulate	notched	Hita Garcia and Fisher 2014	mostly lowland or montane rainforests, rarely dry forests or open habitats
1	***severini***	MAD	11	spatulate	notched	Hita Garcia and Fisher 2014	lowland or montane rainforests
2	***tsingy***	MAD	11	spatulate	notched	Hita Garcia and Fisher 2012a	dry forest
22	***tortuosum***	NEA, AFR, MAD, ORI & INA	11	spatulate	notched	Hita Garcia and Fisher 2012b	predominantly lowland or montane rainforests
1	***weitzeckeri***	AFR & MAL	11	spatulate	notched	Hita Garcia and Fisher 2011	dry forests, savanna, grassland, anthropogenic habitats
3	***bicarinatum***	AFR, ORI, INA & MALT	12	triangular to dentiform	notched	Hita Garcia and Fisher 2011	habitat generalist
1	***obesum***	ORI, INA & MAL, T	12	triangular to dentiform	notched	Hita Garcia and Fisher 2011	habitat generalist
2	***sericeiventre***	AFR & MAL	12	triangular to dentiform	entire	Hita Garcia and Fisher 2011	anthropogenic habitats, spiny forest, thicket, coastal and littoral forests, woodland
1	***setigerum***	AFR & MAD	12	triangular to dentiform	entire	in this study	dry forest
7	***simillimum***	AFR, MAL & MAL, T	12	triangular to dentiform	entire	in preparation	habitat generalist
2	***tosii***	MAD	12	triangular to dentiform	entire	Hita Garcia and Fisher 2011	lowland or montane rainforests
125							

### Identification key to *Tetramorium* species groups in the Malagasy region (workers)

The species group key presented here is based on the one published in Hita Garcia and Fisher (2011). Although the key in that publication still works for most species in Madagascar, it does not accommodate them all. The recent revisions of most species groups, with the establishment of some new groups (Hita Garcia and Fisher 2012a, 2012b, 2014b), require an updated and improved key. The following key applies to the 19 groups we currently recognise, which contains around 125 species (the *Tetramorium ranarum* and *Tetramorium simillimum* groups will be revised in a future publication and the species count for these two groups is temporary).

**Table:** 

1	Species with distinctly branched hairs, usually a mixture of simple, bifid, and trifid hairs (Fig. 2A, B)	***Tetramorium obesum* group**
–	Species without branched hairs; hairs present neither bifid nor trifid, either with simple pilosity (Fig. 2C), or with reduced pilosity but short appressed pubescence (Fig. 2D)	**2**
2	Antennae 12-segmented (Fig. 3A); sting appendage triangular to dentiform, acute apically (Fig. 3B)	**3**
–	Antennae 11-segmented (Fig. 3C); sting appendage spatulate, broadened apically (Fig. 3D)	**7**
3	Anterior clypeal margin with distinct median impression (Fig. 4A)	***Tetramorium bicarinatum* group**
–	Anterior clypeal margin always entire and convex, never with distinct median impression (Fig. 4B, C)	**4**
4	Propodeum armed with long to extremely long spines (PSLI 30–49), at least 2 to 3 times longer than metapleural lobes (Fig. 5A)	***Tetramorium tosii* group**
–	Propodeum either unarmed (Fig. 5B), armed with small triangular teeth or denticles, or armed with medium-sized spines (Fig. 5C), propodeal spines at most only as long or weakly longer than metapleural lobes, more often propodeal spines distinctly shorter than metapleural lobes	**5**
5	Lateral portion of clypeus prominent, raised to a tooth or denticle in full-face view (Fig. 4B); propodeal spines medium-sized and spinose, approximately of same length as metapleural lobes (Fig. 5C)	***Tetramorium sericeiventre* group**
–	Lateral portion of clypeus never modified as above (Fig. 4C); propodeum either unarmed (Fig. 5B) or armed with small triangular teeth or denticles that are shorter than metapleural lobes (Fig. 6E, F)	**6**
6	Head in full-face view relatively thin (CI < 80) and antennal scapes very long (SI > 120) (Fig. 6A); in general appearance head, antennae, and legs elongate and slender (Fig. 6E)	***Tetramorium setigerum* group**
–	Head in full-face view relatively thicker (CI > 85) and antennal scapes conspicuously much shorter (SI < 92) (Fig. 6B, C, D); in general appearance more compact species with thicker heads and shorter antennae and legs (Fig. 6F)	***Tetramorium simillimum* group**
7	Petiolar node and postpetiole strongly squamiform, petiolar node with anterior and posterior faces parallel and well developed, straight dorsum; petiole and postpetiole always completely unsculptured, smooth, and shining; standing pilosity scarce or absent on dorsal mesosoma and waist segments, first gastral tergite without standing pilosity (Fig. 7A)	***Tetramorium weitzeckeri* group**
–	Character combination never as above; petiole and postpetiole variably shaped, especially postpetiole never squamiform as above (Fig. 7B, C, D, E, 8C, D); if petiole squamiform and postpetiole weakly squamiform, then petiolar dorsum reduced and strongly tapering backwards posteriorly (Fig. 7F)	**8**
8	Pronotum anterodorsally with distinct protuberance or bulge (Fig. 8A, B)	***Tetramorium bonibony* group (in part)**
–	Pronotum anterodorsally without any protuberance or bulge (Fig. 8C, D)	**9**
9	First gastral tergite with strongly appressed pubescence of varying length and without any standing hairs (Fig. 9A, B), or with short appressed to erect pilosity without any long, erect to suberect hairs (Fig. 9C)	**10**
–	First gastral tergite usually with long, erect to suberect pilosity (Fig. 9D, E, F)	**17**
10	Antennal scrobes well developed with sharply defined posterior and ventral margins (Fig. 10A, B)	***Tetramorium ranarum* group (in part)**
–	Antennal scrobes usually weakly developed, never with well-defined posterior and ventral margins (Fig. 10C, D, E)	**11**
11	In profile petiolar node rectangular nodiform with sharply angled anterodorsal and posterodorsal margins; both waist segments strongly sculptured (Fig. 11A, B)	**12**
–	Petiolar node rectangular nodiform with conspicuously rounded anterodorsal and/or posterodorsal margins (Fig. 11C), high rounded nodiform (Fig. 11D), or squamiform and strongly anteroposteriorly compressed (Fig. 11E); waist segments usually completely unsculptured, smooth, and shining, rarely with very weak (Fig. 11D, E), superficial sculpture (Fig. 11C)	**13**
12	Propodeum armed with short to moderately long spines (PSLI 18–25); dorsum of head and mesosoma without any standing pilosity (Fig. 12A, B)	***Tetramorium ranarum* group (in part)**
–	Propodeum armed with very long spines (PSLI 50–53); dorsum of head and mesosoma with standing pilosity (Fig. 12C)	***Tetramorium tortuosum* group (in part)**
13	Larger species (HW 0.82–1.03; WL 1.14–1.48); mesosoma comparatively long and slender (LMI 35–37) without distinct margination between lateral and dorsal mesosoma; propodeal spines always very long (PSLI 38–43); body colouration always dark brown to black (Fig. 13A)	***Tetramorium severini* group**
–	Character combination never as above, especially mesosoma either with strong margination between sides and dorsum (Fig. 13B, C, E, F) or relatively shorter, high, and compact (Fig. 13D); usually much smaller species (HW 0.43–0.87; WL 0.54–1.22) with almost always brighter body colour, but if body size in range of above, then either mesosoma clearly higher and more compact (LMI 42–49) (Fig. 13D) or propodeal spines/teeth much shorter (PSLI 12–28) (Fig. 13B)	**14**
14	Dorsum of mesosoma generally completely unsculptured (Fig. 14A), very rarely with few superficial, weak rugulae laterally, but median area of promesonotum always unsculptured and shiny (Fig. 14B, C); mandibles always conspicuously sculptured	**15**
–	Mesosoma usually strongly sculptured (Fig. 14E, F), very rarely with weak rugulose sculpture including median area of promesonotum (Fig. 14D), but then mandibles completely unsculptured and very smooth and shiny	**16**
15	Propodeal spines long and metapleural lobes short (Fig. 15A); petiolar node in dorsal view distinctly wider than long (Fig. 15B)	***Tetramorium bessonii* group (in part)**
–	Propodeal spines/teeth comparatively short and metapleural lobes of almost similar size (Fig. 15C); petiolar node in dorsal view longer than wide (Fig. 15D)	***Tetramorium tsingy* group**
16	Mandibles always unsculptured, smooth, shining (Fig. 16A, B); waist segments always without long, erect to suberect pilosity (Fig. 16E, F)	***Tetramorium schaufussii* group (in part)**
–	Mandibles variably sculptured (Fig. 16C, D); waist segments usually with long, erect to suberect pilosity (Fig. 16G); if pilosity absent (Fig. 16H), then mandibles conspicuously sculptured (Fig. 16D)	***Tetramorium naganum* group (in part)**
17	Antennal scrobes very well developed and distinctly impressed with sharply defined posterior and ventral margins; scrobes with very conspicuous median longitudinal scrobal carina, carina always ending between posterior eye margin and posterior margin of scrobe (Fig. 17A, C); petiolar node in dorsal view always noticeably broader than long (Fig. 17B), and in profile with more or less rounded antero- and posterodorsal margins (Fig. 17C); dorsal mesosoma with longitudinally rugose sculpture (Fig. 17B); all dorsal surfaces of body with abundant, usually dense, long, and suberect to erect hairs (Fig. 17C)	***Tetramorium plesiarum* group**
–	Character combination never as above; usually antennal scrobes either almost absent (Fig. 17D, E) to weakly developed (Fig. 17F), or moderately developed and distinctly impressed, but without sharply defined posterior and ventral margins or a strong median longitudinal carina (Fig. 17G); if antennal scrobes well developed with sharp margin all around and median scrobal carina developed (Fig. 17H, I), then either dorsum of mesosoma reticulate-rugose (Fig. 17J) and/or petiolar node in profile rectangular nodiform with sharply angled antero- and posterodorsal margins (Fig. 17K)	**18**
18	Sculpture on head, mesosoma, and waist segments strongly reduced: head usually very weakly sculptured (especially posteriorly) (Fig. 18A, B, C, D), dorsum of mesosoma generally completely unsculptured (Fig. 18K), very rarely with very few weak, superficial rugulae laterally (Fig. 18L), and waist segments always completely unsculptured	**19**
–	Sculpture never as strongly reduced as above, head always and to a great extent sculptured (Fig. 18E, F, G, H, I, J), dorsum of mesosoma always completely sculptured, (Fig. 18M, N), and waist segments variably sculptured	**20**
19	Mesosoma only weakly marginate between lateral and dorsal mesosoma, instead sides of mesosoma generally rounding more or less smoothly onto the dorsum (Fig. 19A), mesosoma also relatively high and compact (LMI 43–48); mesosoma and first gastral tergite with relatively dense, short to moderately long, appressed to decumbent pubescence intermixed with relatively scarce, much longer erect pilosity (Fig. 19A)	***Tetramorium bessonii* group (in part)**
–	Mesosoma usually with strong margination between lateral and dorsal mesosoma (Fig. 19B), but if mesosoma less marginate (Fig. 19C), then generally more elongate and slender (LMI 37–42); mesosoma and first gastral tergite with few to abundant long, standing hairs, often mixed with substantially fewer and (mostly) shorter appressed to subdecumbent pilosity, pubescence very scarce to absent (Fig. 19B, C)	***Tetramorium marginatum* group (in part)**
20	Relatively large species (HW 0.85–0.97; WL 1.21–1.48); SI relatively high (SI 89–104); propodeal spines very long to extremely long (PSLI 35–68); petiolar node in profile clublike, elongate and longer than high, posterodorsal angle situated higher than anterodorsal (Fig. 20A, B); dorsum of mesosoma conspicuously reticulate-rugose (Fig. 20H); whole body covered by numerous very long, fine, standing hairs (Fig. 20A, B)	***Tetramorium kelleri* group**
–	Character combination never as above, most species much smaller with lower SI and shorter propodeal spines, a differently shaped petiolar node, and with less abundant and shorter pilosity (Fig. 20C, D, E); if species in size range of above and with similarly long propodeal spines and dense, long pilosity, then SI usually lower (SI 65–93), petiolar node more or less rectangular nodiform, and dorsum of mesosoma longitudinally rugose (Fig. 20F, G, I)	**21**
21	Mesosomal outline in profile relatively flat, comparatively low and elongated (LMI 35–39) (Fig. 21A, B, C); in profile petiolar node rounded nodiform to high rounded nodiform with well-rounded margins (Fig. 21A, B), rarely high cuneiform or squamiform (Fig. 21C); propodeum usually with short teeth/denticles and rarely with spines of moderate length (PSLI 7–25, usually below 20); mandibles and waist segments always unsculptured, smooth and shining (Fig. 21A, B, C)	***Tetramorium schaufussii* group (in part)**
–	Character combination never as above; mesosoma usually more compact and higher (LMI usually conspicuously above 40, very rarely below) (Fig. 21D, E, F, G, H), if LMI < 40, then propodeal spines long to very long (PSLI 27–72, usually above 35) and waist segments weakly to conspicuously sculptured (Fig. 21I)	**22**
22	Mesosoma strongly marginate from sides to dorsum (Fig. 21G); dorsum of promesonotum weakly (irregularly) longitudinally rugulose with few, larger unsculptured patches medially, propodeal dorsum either fully unsculptured or only partly irregularly rugulose (Fig. 22A, B); both waist segments always completely unsculptured, smooth and shiny; body colour uniformly yellow (Fig. 21G)	***Tetramorium marginatum* group (in part)**
–	Character combination never as above, especially dorsum of mesosoma usually with conspicuous sculpture along its entire length (Fig. 22C, D, E, F)	**23**
23	Petiolar node in profile dorsally conspicuously anteroposteriorly compressed and strongly narrowing towards apex, giving node a triangular or sharply cuneiform appearance; both waist segments always completely unsculptured, smooth and shiny (Fig. 23A, B, C, D)	**24**
–	Petiolar node variably shaped: rectangular nodiform (Fig. 23E), high nodiform (Fig. 23F), squamiform (Fig. 23G), or broadly/weakly cuneiform (Fig. 23H), in the latter case node not strongly narrowing dorsally and petiole and/or postpetiole distinctly sculptured (Fig. 23H)	**25**
24	Dorsum of mesosoma longitudinally rugose/rugulose (Fig. 24A, B)	***Tetramorium dysalum* group (in part)**
–	Dorsum of mesosoma conspicuously reticulate-rugose, especially anteriorly (Fig. 24C, D, E, F)	***Tetramorium bonibony* group (in part)**
25	Dorsum of mesosoma conspicuously reticulate-rugose throughout its length (Fig. 25A, B, C); petiolar node either squamiform, unsculptured, smooth, and shining or nodiform and usually conspicuously sculptured (Fig. 26A, B, C)	**26**
–	Dorsum of mesosoma longitudinally rugose/rugulose (Fig. 25D, E), sometimes irregularly arranged but still conspicuously longitudinal in nature (Fig. 25F), if the latter, then petiolar node never as above (Fig. 27A, B, C, D, E, F, G, H)	**27**
26	Eyes relatively large (OI 25–26); petiolar node in profile distinctly squamiform and anteroposteriorly compressed (Fig. 26A); waist segments always completely unsculptured, smooth, and shining (Fig. 26A)	***Tetramorium dysalum* group (in part)**
–	Eyes smaller than above, usually significantly so; petiolar node weakly cuneiform to rectangular nodiform and variably sculptured (Fig. 26B, C), but never squamiform and unsculptured as above (Fig. 26B, C)	***Tetramorium ranarum* group (in part)**
27	Petiolar node usually rectangular nodiform with more or less sharply angled anterodorsal and posterodorsal margins (Fig. 27A, B, C), if petiolar node weakly cuneiform (Fig. 27D), then base of first gastral tergite sculptured, at least weakly so; both waist segments always distinctly sculptured, usually distinctly rugose, rarely rugulose (Fig. 27A, B, C, D)	***Tetramorium tortuosum* group (in part)**
–	Petiolar node usually squamiform (Fig. 27E, F), high rounded nodiform (Fig. 27G) or weakly cuneiform (Fig. 27H), if petiolar node weakly cuneiform, then first gastral tergite completely unsculptured, smooth, and shining; petiole and postpetiole fully unsculptured, smooth, and shiny in most species (Fig. 27E, F, G), a few species with one or both waist segments conspicuously sculptured (Fig. 27H)	**28**
28	Pilosity and pubescence on first gastral tergite usually consisting of abundant, long, erect to suberect hairs on top of scarce, much shorter, appressed to decumbent pubescence (Fig. 28A, B, C), very rarely with long decumbent to subdecumbent pilosity on top of scarce appressed pubescence (Fig. 28D)	***Tetramorium dysalum* group (in part)**
–	Pilosity and pubescence on first gastral tergite variable: either with few moderately long, appressed to decumbent pubescence in combination with several much longer, fine, and erect hairs (Fig. 28E), or with a mix of short to moderately long, abundant, decumbent to suberect pilosity, pilosity appearing disorganized due to varying degrees of inclination and hair length (Fig. 28F, G), or with short, abundant, subdecumbent to suberect pilosity, and without any appressed to decumbent pubescence or long, fine erect hairs (Fig. 28H)	***Tetramorium naganum* group (in part)**

**Figure 2.**
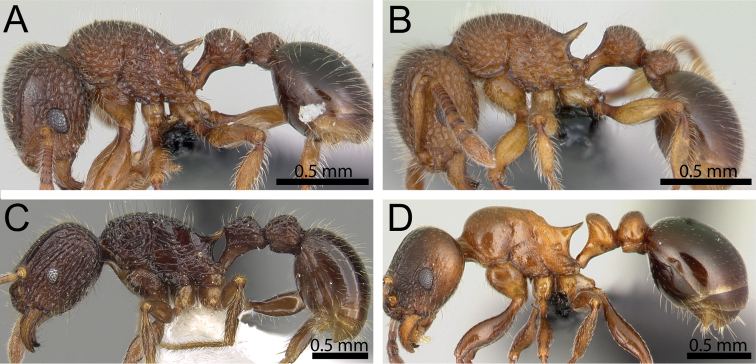
Body in profile. **A, B** *Tetramorium lanuginosum* (CASENT0060515; CASENT0125328) **C** *Tetramorium singletonae* (CASENT0247161) **D** *Tetramorium wardi* (CASENT0475483).

**Figure 3.**
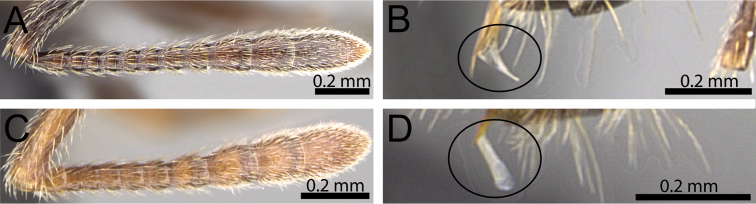
Antennal funiculus and sting appendage (within black ellipses). **A, B** *Tetramorium tosii* (CASENT0249662) **C** *Tetramorium jedi* (CASENT0043578) **D** *Tetramorium hobbit* (CASENT0019207).

**Figure 4.**
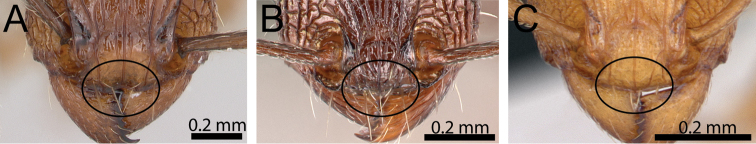
Anterior head in dorsal view (anterior clypeal margin within black ellipses). **A** *Tetramorium bicarinatum* (CASENT0060334) **B** *Tetramorium mahafaly* (CASENT0448984) **C** *Tetramorium simillimum* (CASENT0135001).

**Figure 5.**
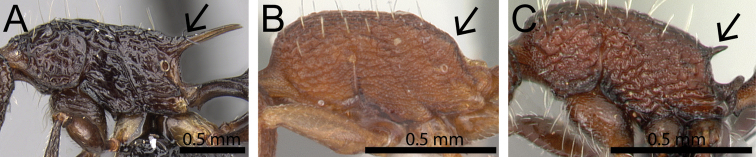
Mesosoma in profile (black arrows indicate propodeal spines/teeth area). **A** *Tetramorium tosii* (CASENT0249662) **B** *Tetramorium anodontion* (CASENT0102334) **C** *Tetramorium mahafaly* (CASENT0449159).

**Figure 6.**
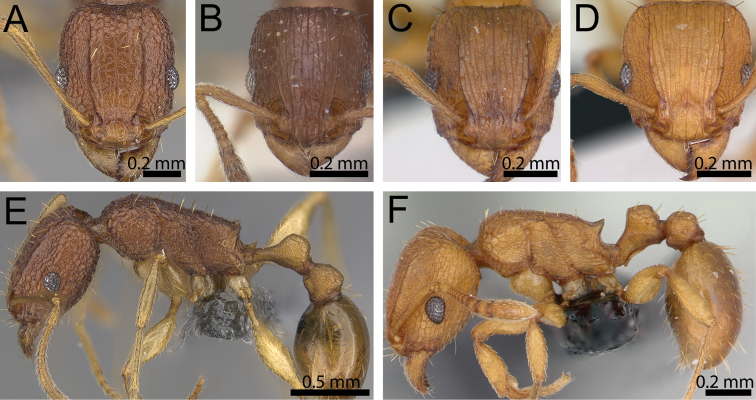
Head in full-face view and body in profile. **A, E** *Tetramorium cavernicola* (CASENT0373132) **B** *Tetramorium scytalum* (CASENT0102337) **C** *Tetramorium caldarium* (CASENT0125225) **D, F** *Tetramorium simillimum* (CASENT0135001).

**Figure 7.**
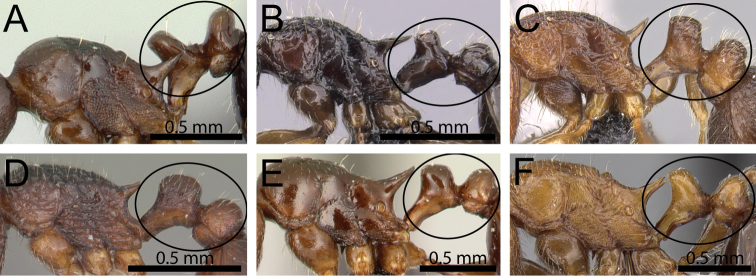
Mesosoma and waist segments in profile (waist segments within black ellipses). **A** *Tetramorium humbloti* (CASENT0134851) **B** *Tetramorium ambatovy* (CASENT0124721) **C** *Tetramorium gollum* (CASENT0074974) **D** *Tetramorium quasirum* (CASENT0102353) **E** *Tetramorium malagasy* (CASENT0449550) **F** *Tetramorium bessonii* (CASENT0247550).

**Figure 8.**
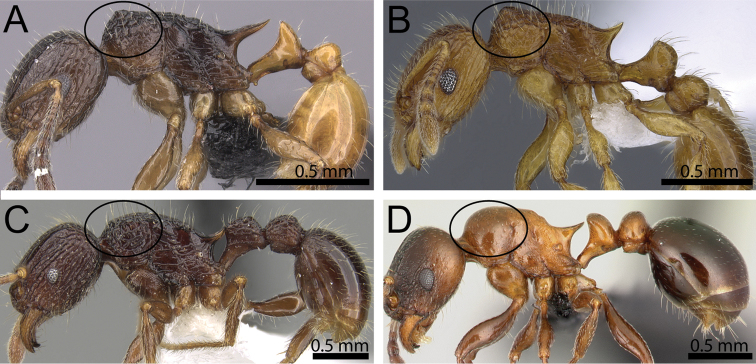
Body in profile (anterodorsal pronotum within black circles). **A** *Tetramorium bonibony* (CASENT0486252) **B** *Tetramorium trafo* (CASENT0404104) **C** *Tetramorium singletonae* (CASENT0247161) **D** *Tetramorium wardi* (CASENT0475483).

**Figure 9.**
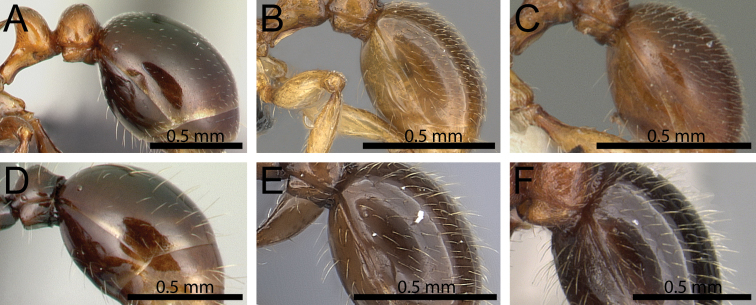
First gastral tergite in profile. **A** *Tetramorium wardi* (CASENT0475483) **B** *Tetramorium cognatum* (CASENT0067891) **C** *Tetramorium naganum* (CASENT0102345) **D** *Tetramorium silvicola* (CASENT0042828) **E** *Tetramorium alperti* (CASENT0042547) **F** *Tetramorium hobbit* (CASENT0019207).

**Figure 10.**
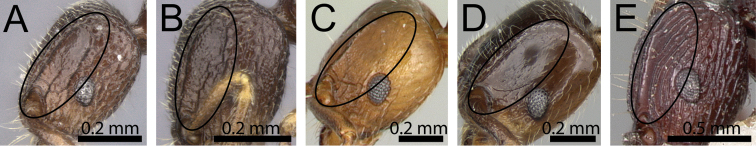
Lateral head in profile (antennal scrobe area within black ellipses). **A** *Tetramorium ibycterum* (CASENT0056460) **B** *Tetramorium* fhg-bilb (CASENT0448625) **C** *Tetramorium artemis* (CASENT0481732) **D** *Tetramorium tyrion* (CASENT0249085) **E** *Tetramorium latreillei* (CASENT0101292).

**Figure 11.**
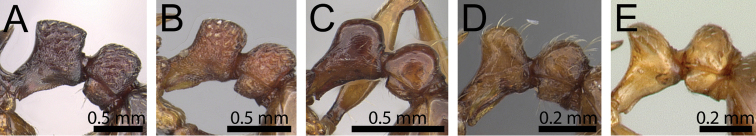
Petiole and postpetiole in profile. **A** *Tetramorium* fhg-ants (CASENT0248302) **B** *Tetramorium* fhg-anub (CASENT0404158) **C** *Tetramorium nassonowii* (CASENT0195504) **D** *Tetramorium naganum* (CASENT0280584) **E** *Tetramorium artemis* (CASENT0481732).

**Figure 12.**
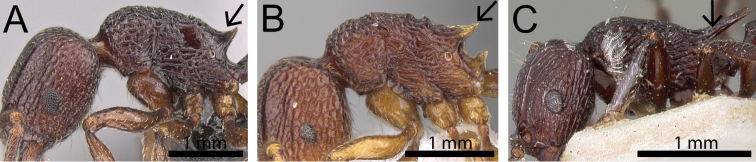
Posterior head and mesosoma in profile (black arrows indicate propodeal spines/teeth. **A** *Tetramorium* fhg-ants (CASENT0248302) **B** *Tetramorium* fhg-anub (CASENT0404158) **C** *Tetramorium latreillei* (CASENT0101292).

**Figure 13.**
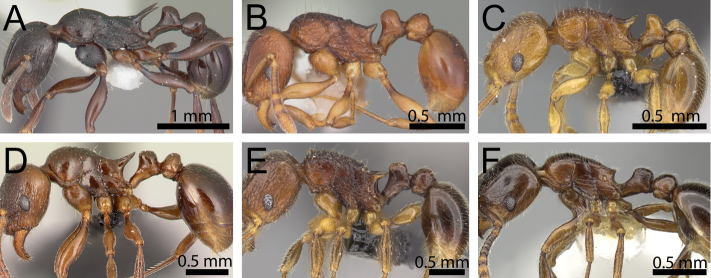
Body in profile. **A** *Tetramorium severini* (CASENT0102397) **B** *Tetramorium proximum* (CASENT0102342) **C** *Tetramorium rumo* (CASENT0073025) **D** *Tetramorium malagasy* (CASENT0449550) **E** *Tetramorium dalek* (CASENT0038402) **F** *Tetramorium tyrion* (CASENT0249085).

**Figure 14.**
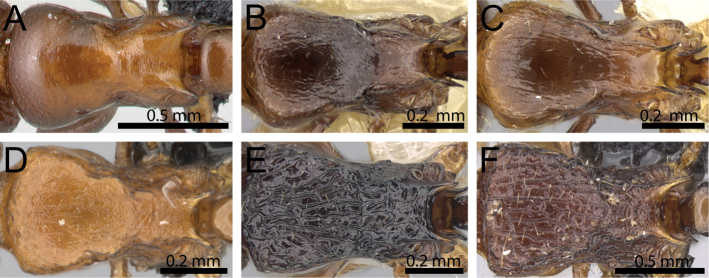
Mesosoma in dorsal view. **A** *Tetramorium wardi* (CASENT0475483) **B** *Tetramorium tyrion* (CASENT0249085) **C** *Tetramorium tsingy* (CASENT0426807) **D** *Tetramorium freya* (CASENT0466944) **E** *Tetramorium camelliae* (CASENT0247496) **F** *Tetramorium dalek* (CASENT0038402).

**Figure 15.**
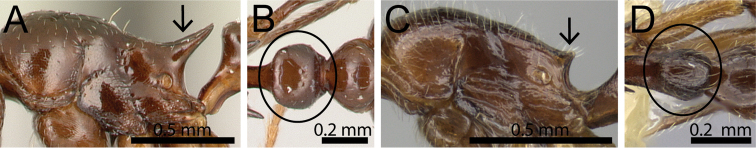
Mesosoma in profile (black arrows indicating propodeal spines/teeth) and petiole in dorsal view (within black ellipse). **A, B** *Tetramorium malagasy* (CASENT0449550) **C, D** *Tetramorium tyrion* (CASENT0249085).

**Figure 16.**
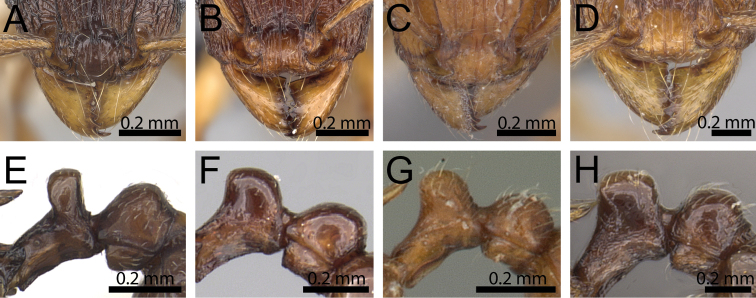
Anterior head in dorsal view and waist segments in profile. **A, E** *Tetramorium camelliae* (CASENT0247496) **B, F** *Tetramorium myrmidon* (CASENT0028635) **C, G** *Tetramorium naganum* (CASENT0102345) **D, H** *Tetramorium dalek* (CASENT0038402).

**Figure 17.**
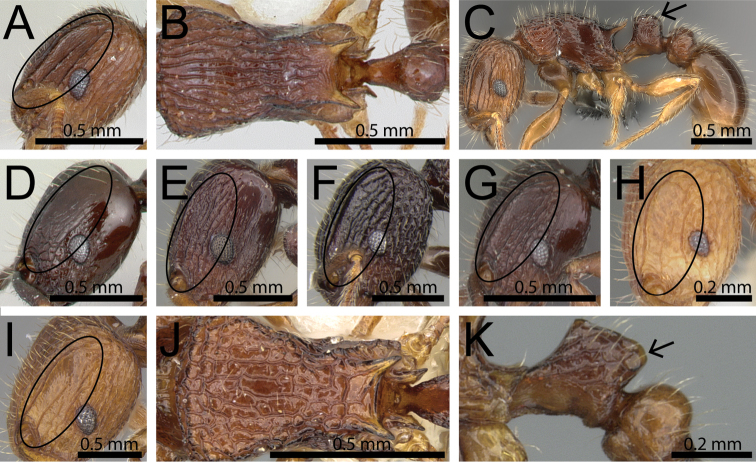
Head (without mandibles) in profile (antennal scrobe area within black ellipses); mesosoma in dorsal view; body and waist segments in profile. **A, B** *Tetramorium plesiarum* (CASENT0172831) **C** *Tetramorium mars* (CASENT0474279) **D** *Tetramorium silvicola* (CASENT0042828) **E** *Tetramorium nassonowii* (CASENT0195504) **F** *Tetramorium adamsi* (CASENT0247296) **G** *Tetramorium dysalum* (CASENT0102348) **H, J, K** *Tetramorium zenatum* (CASENT0102355; CASENT0344941) **I** *Tetramorium* fhg-vazi (CASENT0422522).

**Figure 18.**
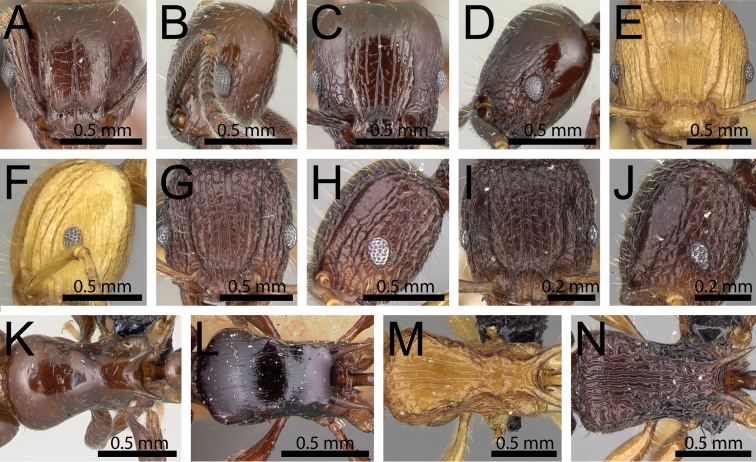
Head (without mandibles) in dorsal and lateral view; Mesosoma in dorsal view. **A, B** *Tetramorium ryanphelanae* (CASENT0454495) **C, D** *Tetramorium silvicola* (CASENT0042828) **E, F, M** *Tetramorium elf* (CASENT0045788) **G, H, N** *Tetramorium jedi* (CASENT0043578) **I, J** *Tetramorium quasirum* (CASENT0280585) **K** *Tetramorium ryanphelanae* (CASENT0454495) **L** *Tetramorium marginatum* (CASENT0101287).

**Figure 19.**
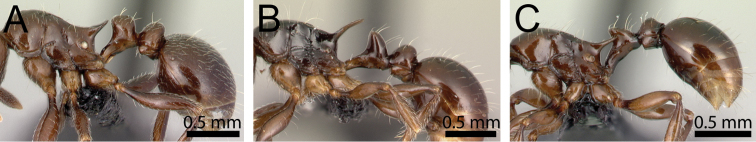
Mesosoma, waist segments, and first gastral tergite in profile. **A** *Tetramorium ryanphelanae* (CASENT0454495) **B** *Tetramorium valky* (CASENT0496394) **C** *Tetramorium silvicola* (CASENT0042828).

**Figure 20.**
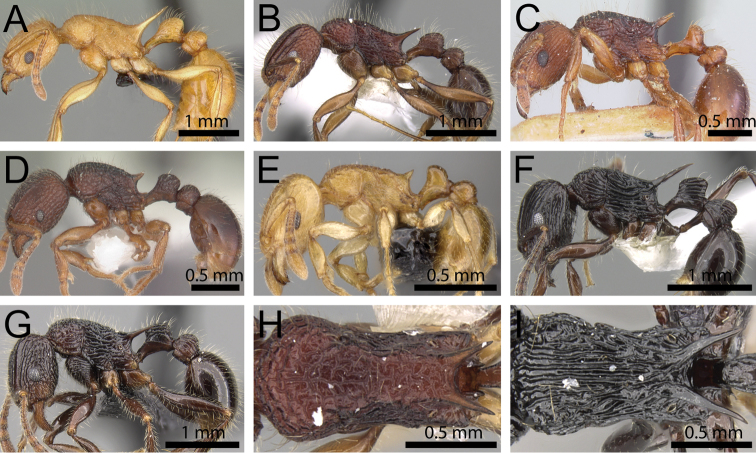
Mesosoma and waist segments in profile; mesosoma in dorsal view. **A** *Tetramorium kelleri* (CASENT0467063) **B, H** *Tetramorium ankarana* (CASENT0247543) **C** *Tetramorium steinheili* (CASENT0101258) **D** *Tetramorium ranarum* (CASENT0102392) **E** *Tetramorium rala* (CASENT0162115) **F** *Tetramorium ambanizana* (CASENT0189238) **G** *Tetramorium nazgul* (CASENT0028625) **I** *Tetramorium smaug* (CASENT0121244).

**Figure 21.**
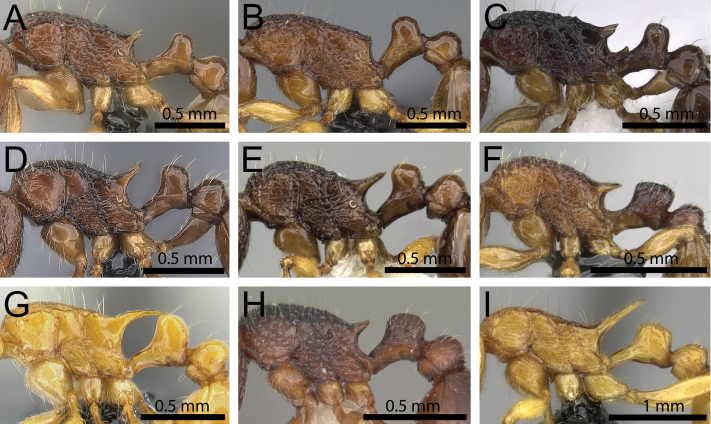
Mesosoma and waist segments in profile. **A** *Tetramorium pseudogladius* (CASENT0153605) **B** *Tetramorium merina* (CASENT0437226) **C** *Tetramorium scutum* (CASENT0189116) **D** *Tetramorium alperti* (CASENT0042547) **E** *Tetramorium vohitra* (CASENT0189167) **F** *Tetramorium* fhg-forc (CASENT0150949) **G** *Tetramorium shamshir* (CASENT0467696) **H** *Tetramorium quasirum* (CASENT0102353) **I** *Tetramorium elf* (CASENT0045788).

**Figure 22.**
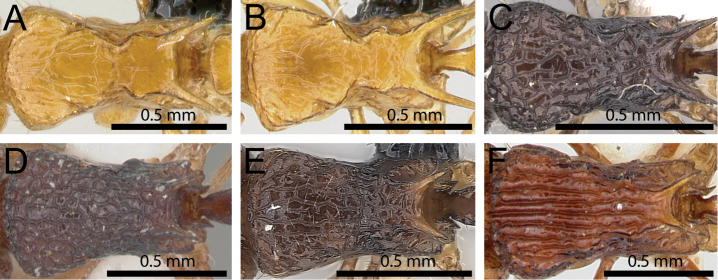
Mesosoma in dorsal view. **A** *Tetramorium norvigi* (CASENT0489037) **B** *Tetramorium shamshir* (CASENT0467696) **C** *Tetramorium nosybe* (CASENT0422207) **D** *Tetramorium ranarum* (CASENT0102392) **E** *Tetramorium alperti* (CASENT0042547) **F** *Tetramorium isectum* (CASENT0172829).

**Figure 23.**
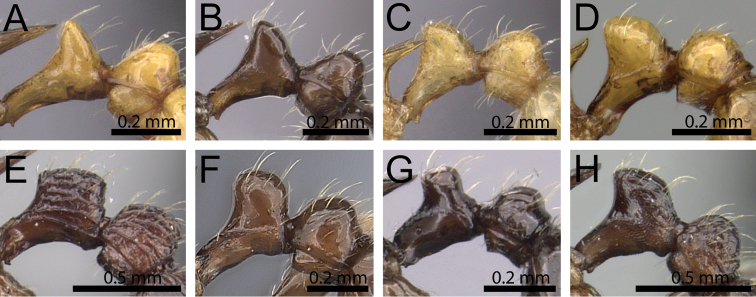
Petiole and postpetiole in profile. **A** *Tetramorium sada* (CASENT0443274) **B** *Tetramorium nosybe* (CASENT0422207) **C** *Tetramorium olana* (CASENT0044485) **D** *Tetramorium mackae* (CASENT0189093) **E** *Tetramorium aherni* (CASENT0045755) **F** *Tetramorium alperti* (CASENT0042547) **G** *Tetramorium ambatovy* (CASENT0124721) **H** *Tetramorium avaratra* (CASENT0445167).

**Figure 24.**
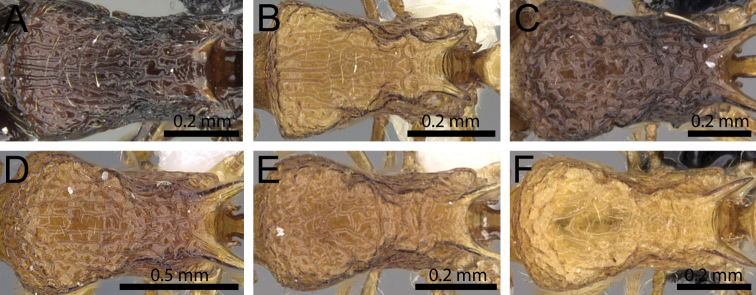
Mesosoma in dorsal view. **A** *Tetramorium orc* (CASENT0487093) **B** *Tetramorium mackae* (CASENT0189093) **C** *Tetramorium sada* (CASENT0443274) **D** *Tetramorium vony* (CASENT0404310) **E** *Tetramorium kali* (CASENT0235221) **F** *Tetramorium olana* (CASENT0044485).

**Figure 25.**
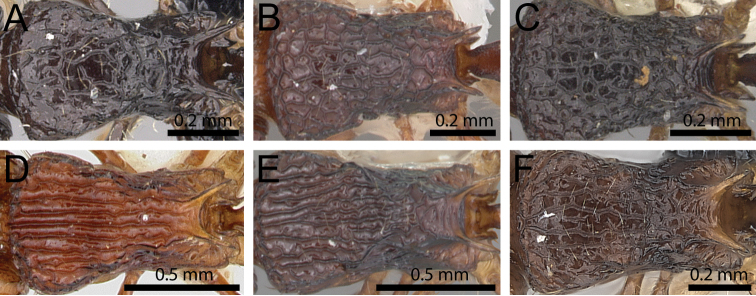
Mesosoma in dorsal view. **A** *Tetramorium ambatovy* (CASENT0124721) **B** *Tetramorium quasirum* (CASENT0102353) **C** *Tetramorium coillum* (CASENT0235219) **D** *Tetramorium isectum* (CASENT0172829) **E** *Tetramorium steinheili* (CASENT0102394) **F** *Tetramorium alperti* (CASENT0042547).

**Figure 26.**
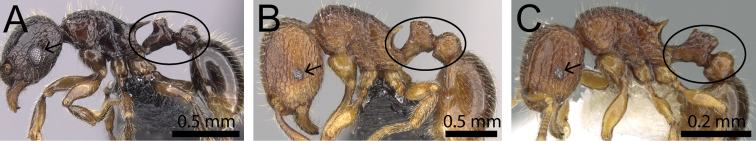
Body in profile (black arrows indicate eyes; wait segments within black ellipses). **A** *Tetramorium ambatovy* (CASENT0124721) **B** *Tetramorium* fhg-mogw (CASENT0056452) **C** *Tetramorium zenatum* (CASENT0344941).

**Figure 27.**
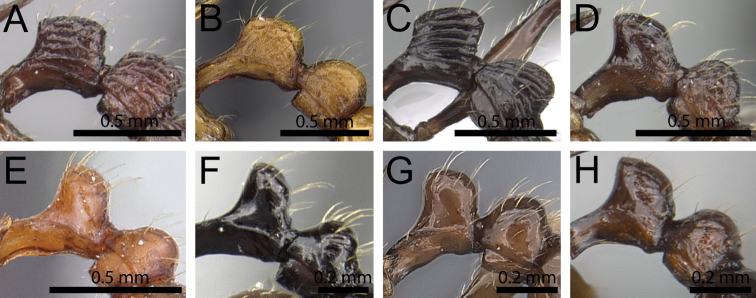
Petiole and postpetiole in profile. **A** *Tetramorium aherni* (CASENT0045755) **B** *Tetramorium voasary* (CASENT0247162) **C** *Tetramorium ambanizana* (CASENT0189238) **D** *Tetramorium avaratra* (CASENT0445167) **E** *Tetramorium steinheili* (CASENT0101258) **F** *Tetramorium yammer* (CASENT0042832) **G** *Tetramorium alperti* (CASENT0042547) **H** *Tetramorium dysalum* (CASENT0037931).

**Figure 28.**
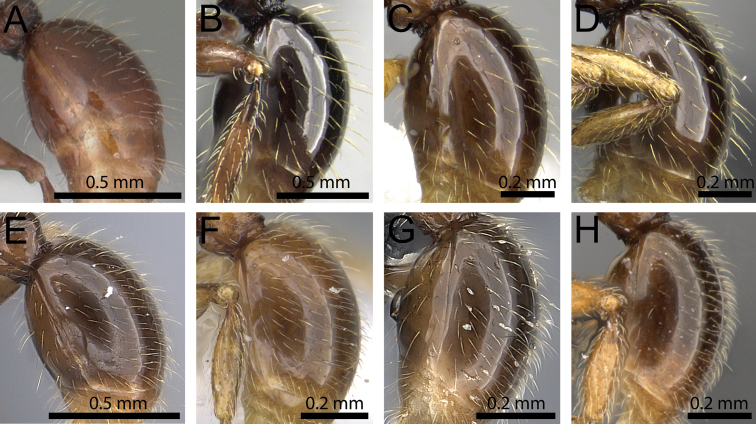
First gastral tergite in profile. **A** *Tetramorium dysalum* (CASENT0102349) **B** *Tetramorium yammer* (CASENT0042832) **C** *Tetramorium vohitra* (CASENT0189167) **D** *Tetramorium sargina* (CASENT0487390) **E** *Tetramorium alperti* (CASENT0042547) **F** *Tetramorium gilgamesh* (CASENT0247312) **G** *Tetramorium gilgamesh* (CASENT0163251) **H** *Tetramorium enkidu* (CASENT0045673).

## Revision of the *Tetramorium setigerum* species group in Madagascar

### Synopsis of the *Tetramorium setigerum* species group in Madagascar

*Tetramorium cavernicola* Hita Garcia & Fisher, sp. n.

### Diagnosis of the *Tetramorium setigerum* group in Madagascar

Twelve-segmented antennae; antennal scapes very long (SI 120–123); anterior clypeal margin entire and clearly convex; frontal carinae well-developed, ending at or approaching posterior head margin; eyes moderate (OI 23–26); anterior face of mesosoma weakly developed, no distinct margination between lateral and dorsal mesosoma; propodeum armed with short triangular to elongate-triangular teeth (PSLI 7–11), propodeal lobes moderately developed, triangular to elongate-triangular, slightly longer and broader than propodeal teeth; petiolar node relatively small, nodiform, with weakly angled anterodorsal and posterodorsal margins, and comparatively long peduncle, petiolar dorsum flat to very weakly convex, node in profile between 1.2 to 1.4 times higher than long (LPeI 73–79), node in dorsal view between 1.2 to 1.3 times longer than wide (DPeI 121–127); postpetiole in profile approximately globular, around 1.0 to 1.1 times higher than long (LPpI 90–98); mandibles striate; clypeus longitudinally rugose/rugulose with well-developed median ruga and usually one or two weaker, sometimes irregular, lateral rugae/rugulae on each side; sculpture on cephalic dorsum irregularly longitudinally rugose to reticulate-rugose; mesosoma laterally irregularly rugulose, dorsally reticulate-rugulose to irregularly rugulose; petiole and postpetiole conspicuously rugulose; ground sculpture on mesosoma and waist segments distinctly reticulate-punctate, much weaker on head; gaster unsculptured, smooth, and shiny; all dorsal surfaces of body with short to moderately long, thick, and apically blunt pilosity; sting appendage triangular to dentiform.

### Taxonomic and biogeographic notes on the group

Prior to this study, the *Tetramorium setigerum* species group appeared endemic to the Afrotropical region where it is widely distributed. Of the 13 species recognised by Bolton (1980), most are found in more arid areas of eastern and southern Africa, a few are distributed in the rainforests of Central Africa, while two species are also found in Ethiopia and the southwestern Arabian Peninsula. The recent finding of *Tetramorium cavernicola* in Madagascar was unexpected since there was no previous indication of the presence of the group on Madagascar or any of the surrounding islands of the South West Indian Ocean. However, as outlined above, considering the strong biogeographical affinities of the *Tetramorium* ant fauna of Madagascar with the Afrotropical region, this is less of a surprise. Indeed, the *Tetramorium setigerum* group has its highest abundance and diversity in South and Southeast Africa, which is geographically comparatively close to Madagascar. As outlined above, other species or species groups that made it from Africa to Madagascar are often of predominantly eastern and southern African origin; examples include *Tetramorium humbloti* from the *Tetramorium weitzeckeri* group and *Tetramorium delagoense* from the *Tetramorium simillimum* group.

**Figure 29.**
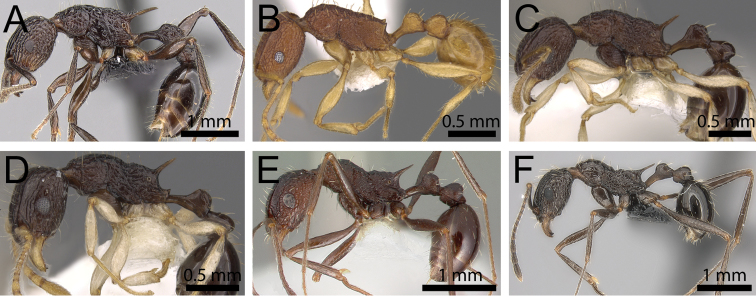
Body in profile. **A** *Tetramorium tosii* (CASENT0249662) **B** *Tetramorium cavernicola* (CASENT0247028) **C** *Tetramorium metactum* (CASENT0901193) **D** *Tetramorium youngi* (CASENT0901192) **E** *Tetramorium dolichosum* (CASTYPE13388) **F** *Tetramorium perlongum* (CASENT0135293).

The *Tetramorium setigerum* group cannot be mistaken for any other Malagasy species group. Its possession of twelve-segmented antennae, an entire and convex clypeal margin, and simple pilosity distinguish it from most other groups, except the *Tetramorium sericeiventre*, *Tetramorium simillimum*, and *Tetramorium tosii* groups. In the *Tetramorium sericeiventre* group the clypeus is distinctly modified, with the lateral portion being very prominent and raised into a tooth/denticle in full-face view while the clypeus of the *Tetramorium setigerum* group lacks such a tooth/denticle. Also, the species of the *Tetramorium simillimum* group possess much shorter antennal scapes (SI always much shorter than 100) than the *Tetramorium setigerum* group (SI over 120). The differentiation of the latter from the *Tetramorium tosii* group is more problematic. Despite the fact that the only representative of the *Tetramorium setigerum* group in Madagascar and the two species of the *Tetramorium tosii* group are easily separable (see key couplets 4 to 6), only a few morphological characters separate both groups if one also considers all members of the *Tetramorium setigerum* group from the Afrotropical region. Nevertheless, we prefer to keep both groups separate for the following reasons. First, the shape of the petiolar node is low, elongate, clublike, and always longer than high in the *Tetramorium tosii* group (Fig. 29A), whereas it is variably nodiform in the *Tetramorium setigerum* group, but usually higher than long and never low and elongate (Fig. 29B, C, D, E, F). Second, the standing pilosity in the *Tetramorium tosii* group consists of long, fine, acute hairs (Fig. 29A), whereas the pilosity in most members of the *Tetramorium setigerum* group is thick, short to moderately long, and usually blunt apically (Fig. 29B, E, F). Nevertheless, this is not the case in *Tetramorium metactum* Bolton and *Tetramorium youngi* Bolton since they have long and fine pilosity (Fig. 29C, D). This may seem contradictory, but leads to our next argument. Third, we strongly suspect that the *Tetramorium setigerum* group is not a monophyletic group, but might be composed of different lineages that share a number of morphological characters that have evolved convergently. For example, the morphology of *Tetramorium cavernicola* from Madagascar is certainly closer to the species complex around *Tetramorium setigerum* Mayr and allies (Bolton 1980) than to *Tetramorium metactum* or *Tetramorium youngi*. In sum, the relationships between the *Tetramorium tosii* and the *Tetramorium setigerum* groups, as well as within the latter group, remain unclear, and we prefer to keep the groups as they are until additional data can provide better resolution of the groupings.

#### Tetramorium cavernicola

Taxon classificationAnimaliaHymenopteraFormicidae

Hita Garcia & Fisher sp. n.

http://zoobank.org/FBE17724-6130-4C3B-8790-1C60C7278D62

**Figure 30.**
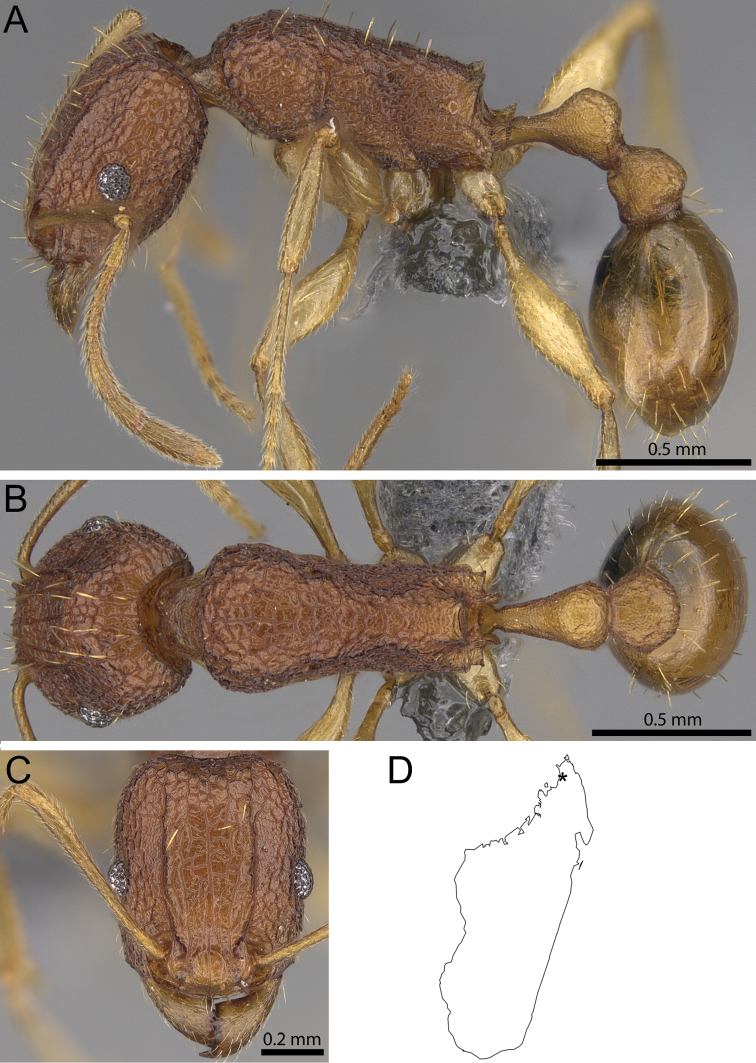
*Tetramorium cavernicola* sp. n. paratype (CASENT0373132). **A** Body in profile **B** Body in dorsal view **C** head in full-face view **D** map of Madagascar showing the type locality (black star symbol).

Figs 6A, E , 29B , 30

##### Type material.

**Holotype**, pinned worker, MADAGASCAR, Antsiranana, Réserve Spéciale d’Ankarana, Andrafiabe, 12.92968 S, 49.05983 E, 59 m, in cave, ground nest, collection code BLF32473, 26.XI.2013 (*B. Fisher et al.*) (CASC: CASENT0247028). **Paratypes**, 15 pinned workers with same data as holotype (CASC: CASENT0247022; CASENT0247023; CASENT0247024; CASENT0247025; CASENT0247026; CASENT0247027; CASENT0247028; CASENT0247357; CASENT0247358; CASENT0248742; CASENT0248745; CASENT0248746; CASENT0373132; HLMD: CASENT0247029; MHNG: CASENT0248743; NHMB: CASENT0248744); and three pinned workers with same data as holotype except collection code BLF32472 and collected as ground foragers (BMNH: CASENT0247021; CASC: CASENT0247020; MCZ: CASENT0373133).

##### Non-type material.

MADAGASCAR: Antsiranana, Réserve Spéciale d’Ankarana, Andrafiabe, 12.92968 S, 49.05983 E, 59 m, 26.XI.2013 (*B. Fisher et al.*).

##### Diagnosis.

*Tetramorium cavernicola* differs from all other Malagasy congeners by the following combination of characters: 12-segmented antennae; anterior clypeal margin entire and convex; lateral clypeus not modified into tooth or denticle; antennal scape very long (SI 120–123); mesosoma in profile relatively low and slender (LMI 35–36); and propodeum armed with very short teeth/spines (PSLI 7–11).

##### Worker measurements

**(N=15).** HL 0.74–0.78 (0.76); HW 0.58–0.61 (0.60); SL 0.71–0.75 (0.72); EL 0.14–0.15 (0.14); PH 0.32––0.35 (0.33); PW 0.45–0.48 (0.46); WL 0.92–0.99 (0.95); PSL 0.06–0.08 (0.07); PTL 0.17–0.19 (0.18); PTH 0.22–0.24 (0.23); PTW 0.21–0.23 (0.22); PPL 0.22–0.24 (0.23); PPH 0.24–0.25 (0.25); PPW 0.26–0.28 (0.27); CI 77–79 (78); SI 120–123 (122); OI 23–26; DMI 47–50 (49); LMI 35–36 (35); PSLI 7–11 (9); PeNI 47–49 (47); LPeI 73–79 (77); DPeI 121–127 (123); PpNI 56–60 (58); LPpI 90–98 (94); DPpI 113–123 (117); PPI 120–127 (124).

##### Worker description.

Head much longer than wide (CI 77–79); posterior head margin weakly to moderately concave. Anterior clypeal margin entire and convex. Frontal carinae strongly developed, moderately raised, usually becoming weaker after posterior eye level, approaching or ending at posterior head margin; antennal scrobes very weak to absent. Antennal scapes very long, weakly surpassing posterior head margin (SI 120–123). Eyes moderately large (OI 23–26). Mesosomal outline in profile relatively flat, elongate and low (LMI 35–36), weakly marginate from lateral to dorsal mesosoma; promesonotal suture and metanotal groove absent. Propodeum armed with short, triangular teeth (PSLI 7–11), propodeal lobes moderately developed, triangular to elongate-triangular, slightly longer and broader than propodeal teeth. Petiolar node nodiform with moderately rounded antero- and posterodorsal margins, in profile between 1.2 and 1.4 times higher than long (LPeI 73–79), anterior and posterior faces not parallel, node weakly narrowing towards dorsum, anterodorsal and posterodorsal margins situated at about same height and both weakly to moderately angled, petiolar dorsum flat to very weakly convex; node in dorsal view around 1.2 to 1.3 times wider than long (DPeI 121–127), in dorsal view pronotum around 2.0 to 2.1 times wider than petiolar node (PeNI 47–49). Postpetiole in profile approximately globular, around 1.0 to 1.1 times higher than long (LPpI 90–98); in dorsal view around 1.1 and 1.2 times wider than long (DPpI 113–123), pronotum around 1.7 to 1.8 times wider than postpetiole (PpNI 56–60). Postpetiole in profile appearing distinctly more voluminous than petiolar node, postpetiole in dorsal view around 1.2 to 1.3 times wider than petiolar node (PPI 120–127). Mandibles striate; clypeus longitudinally rugose/rugulose with well-developed median ruga and usually one or two weaker, sometimes irregular, lateral rugae/rugulae on each side; cephalic dorsum between frontal carinae anteriorly towards posterior clypeal margin with three or four distinct but irregularly shaped longitudinal rugae with numerous cross-meshes, halfway between eye level and posterior head margin fluent transition to well-developed rugoreticulum ranging to posterior head margin; scrobal area only weakly sculptured, remainder of lateral head clearly reticulate-rugose. Mesosoma laterally and dorsally conspicuously reticulate-rugose; forecoxae unsculptured, smooth, and shining. Petiole and postpetiole irregularly rugulose, better developed on dorsum than sides. First gastral tergite unsculptured, smooth, and shiny. Ground sculpture on cephalic dorsum between frontal carinae weak, distinctly reticulate-punctate on lateral head, mesosoma, and waist segments, absent from gaster. All dorsal surfaces of body with short to moderately long, thick, and apically blunt pilosity; appressed pubescence on first gastral tergite strongly reduced to absent. Anterior edges of antennal scapes and dorsal (outer) surfaces of hind tibiae with decumbent to suberect hairs. Head and mesosoma reddish brown; waist segments lighter in colour, usually orange brown; mandibles, antennae, and legs yellowish brown.

##### Etymology.

The name of the new species is a Latin noun and means “cave dweller” or “cave inhabitant”. It refers to the microhabitat where the type series was collected. The species epithet is a nominative noun in apposition.

##### Distribution and biology.

Currently, *Tetramorium cavernicola* is only known from Ankarana (Fig. 30D), where it was collected from a cave. The collection locality and the fact that the species is not known from outside the cave imply that *Tetramorium cavernicola* might be a specialised, cave-adapted ant. The generally very slender body and very long antennae and legs also support cave specialisation. Nevertheless, we do not consider the new species an obligate cave inhabitant. Arthropods that have evolved a cave-obligate lifestyle usually have a distinct set of morphological adaptations: reduction or loss of eyes, pigments, and wings; thinning of the cuticle; elongate antennae and legs; and slender body (Christiansen 1962; Culver 1982; Barr 1985). Yet the eyes, pigment, and wings in *Tetramorium cavernicola* are clearly not reduced since its eyes are always well developed, as are the wings in the queen and male castes, and the body colouration is brownish. In addition, we cannot detect any thinning of the cuticle. The slender gestalt and long antennae and legs could argue for cave adaptation, but are actually very typical of most species in the *Tetramorium setigerum* group. The antennae and legs of *Tetramorium dolichosum* Bolton and *Tetramorium perlongum* Santschi (Fig. 29E, F) are much longer than in *Tetramorium cavernicola*, even though these species do not live in caves. *Tetramorium cavernicola* appears to nest in the ground since most of the type series was collected from a ground nest, but no additional natural history data exists for this species.

##### Discussion.

*Tetramorium cavernicola* is a very distinctive element of the Malagasy *Tetramorium* fauna and cannot be mistaken for any other congener based on the diagnosis provided above. There are some morphological similarities to the two species of the *Tetramorium tosii* group, as outlined earlier, but the distinction between these is easily found by comparing the shape of the head, the length of the antennal scapes, and the propodeal spines. In *Tetramorium cavernicola* the head is noticeably thinner (CI 77–79) and the antennal scapes are much longer (SI 120–123) while the propodeal spines are reduced to short teeth (PSLI 7–11). By contrast, the species in the *Tetramorium tosii* group have a thicker head (CI 85–91), much shorter scapes (SI 79–104), and much longer propodeal spines (PSLI 30–49).

##### Variation.

Since *Tetramorium cavernicola* is only known from the type locality, there is no observable intraspecific variation.

## Supplementary Material

XML Treatment for Tetramorium cavernicola
